# Effects of Paliperidone Palmitate on Coagulation: An Experimental Study

**DOI:** 10.1155/2014/964380

**Published:** 2014-02-09

**Authors:** Enver Demirel Yılmaz, Sedat Motor, Fatih Sefil, Neslihan Pınar, Hanifi Kokacya, Mustafa Kisa, Suleyman Oktar

**Affiliations:** ^1^Department of Psychiatry, Faculty of Medicine, Bezmialem University, Istanbul, Turkey; ^2^Department of Biochemistry, Faculty of Medicine, Mustafa Kemal University, Hatay, Turkey; ^3^Department of Physiology, Faculty of Medicine, Mustafa Kemal University, Hatay, Turkey; ^4^Department of Pharmacology, Faculty of Medicine, Mustafa Kemal University, Hatay, Turkey; ^5^Department of Psychiatry, Faculty of Medicine, Mustafa Kemal University, Hatay, Turkey; ^6^Department of Pharmacology, Faculty of Medicine, Mevlana University, Konya, Turkey

## Abstract

*Objective*. The aim of the present study was to examine the effects of a new antipsychotic drug paliperidone palmitate on hemogram and coagulation parameters in rats. *Materials and Methods*. Experiments were performed on 22 female albino Wistar rats (8–12 weeks old). Control group was given drinking water as vehicle (0.3 mL). PAL-1 rats were given 1 mg/kg paliperidone palmitate (in 0.3 mL drinking water) by oral gavage once a day for ten days and PAL-3 rats received 3 mg/kg paliperidone palmitate (in 0.3 mL drinking water) by oral gavage for ten days. Blood samples were drawn from the heart 24 hours after the last drug dose, and hemogram and coagulation parameters were measured with automated analyzers. *Results*. Hemogram did not change in the paliperidone treated groups compared to the controls. Factor VIII levels decreased in the PAL-1 and PAL-3 groups; and this decrease was significantly greater in the PAL-3. Factor IX levels decreased in PAL-3 rats, but its levels also increased in PAL-1 rats compared to the control. *Discussion*. Paliperidone has led to changes in the serum levels of coagulation factors VIII and IX in rats. As a result, paliperidone may be causing thromboembolism or bleeding in a dose-independent manner.

## 1. Introduction

Several studies showed the relationship between venous thromboembolism and the use of antipsychotic drugs especially atypical agents [1]. Many clinical studies have pointed to thrombogenic potential of atypical antipsychotic compounds such as clozapine, risperidone, and olanzapine. Cerebrovascular adverse events such as stroke and transient ischemic attack were observed at a high rate in some clinical trials in patients who received treatment with olanzapine or risperidone [2]. For example, the incidence of cerebrovascular adverse events related to risperidone in elderly patients was 3.8%, compared with 1.5% placebo [3]. In a previous study, the relationship between antipsychotic medications and treatment of venous thromboembolism was strongly supported by a large, nested case-control study [4]. Although several hypotheses have been proposed, the biological mechanism explaining this relationship is unknown. Drug-induced sedation, obesity, hyperleptinemia, antiphospholipid antibodies, and increased activity of the hemostatic system may be high risk for thromboembolism [5].

Paliperidone (INVEGA, Ortho-McNeil-Janssen Pharmaceuticals, Inc., Titusville, NJ, USA) was approved by the FDA for the treatment of schizophrenia in 2006. Paliperidone palmitate, a long-acting atypical antipsychotic drug, is used for adults for the treatment of schizophrenia. Paliperidone is the 9-OH metabolite of risperidone and paliperidone palmitate is also the palmitate ester of paliperidone. Paliperidone palmitate belongs to the chemical class of benzisoxazole derivatives [6]. The precise mechanism of paliperidone is not yet known. The mechanism of action of paliperidone is attributed to the antagonism of brain dopamine D_2_ and serotonin 5-HT_2A_ receptors [7, 8]. Paliperidone is minimally metabolized in the liver [8]. As mentioned above, paliperidone is not metabolized extensively by the liver. Invega application file of FDA reported that drug-related changes in hematology and clinical chemistry were small, and but not dose-dependent [9]. As of today, there are no studies in the literature investigating the relationship between paliperidone and hemostasis.

There is a need to investigate the association between the use of paliperidone and hemostatic abnormalities. The purpose of the present study was to examine the effects of paliperidone on hemogram and especially coagulation parameters in an experimental manner.

## 2. Materials and Methods

All the animal experiment protocols were approved by the Animal Ethical Committee of Mustafa Kemal University. Experiments were performed on 22 female albino Wistar rats (8–12 weeks old) which were obtained from the experimental research center of Mustafa Kemal University. Rats were kept in a room maintained at ambient temperature and humidity (25 ± 5°C, 55 ± 5%) under a day/night regime (day 8:00–20:00 and night 20:00–8:00) and allowed a commercial standard rat diet and water ad libitum. Rats were weighed before the experiment and those weighing 200–300 g were divided randomly into three groups.

A total of 8 rats were involved in the first group, which was also the control group, and rats were given drinking water as vehicle (0.3 mL). A total of 7 rats were gathered as the second group, receiving 1 mg/kg paliperidone palmitate (in 0.3 mL drinking water) orally (gavage) once a day for ten days (called PAL-1 group); the third group of 7 rats received 3 mg/kg paliperidone palmitate (in 0.3 mL drinking water) orally once a day for ten days (called PAL-3 group). One paliperidone palmitate tablet (marketed as Invega) was powdered and prepared at an appropriate concentration by diluting with drinking water. Twenty-four hours after the last drug administration, general anesthesia with ketamine (50 mg/kg) and xylazine (3 mg/kg) was administered intraperitoneally to rats. Then the blood of rats was drawn by a syringe from the hearts of rats. Rats were killed at the end of blood-taking process. A portion of the blood samples was put into tubes containing sodium citrate 3.8% (4.5 mL blood sample and 0.5 mL citrate mixture) for coagulation analysis. The other portion of blood samples was put into hemogram tubes. Plasma samples were taken by centrifuge at 3000 g for ten minutes. Hemogram was measured by an auto hematology analyzer (Mindray, BC-6800, Shenzhen, China). Hemostatic parameters were measured by an automatic coagulation analyzer (MDA-II automated coagulation analyzer system, bioMerieux Inc., USA). Normal reference range was PT: 10–15 sec, aPTT: 23–38 sec, and INR: 0.8–1.2 sec. Coagulation factors, protein C, protein S, and AT-III were evaluated by automated analyzer system (AMAX-200 automated analyzer, Trinity Biotech, Ireland).

Data was analyzed by using a commercially available statistics software package (SPSS for Windows v. 15.0, Chicago, USA). Distribution of the groups was analyzed with a one-sample Kolmogorov-Smirnov test. Groups showed normal distribution, so that parametric statistical methods were used to analyze the data. One-way ANOVA test was performed and post hoc multiple comparisons were made using least-squares differences. Mann-Whitney *U* test was used for the groups with abnormal distribution. Results are presented as mean ± SD. *P* < 0.05 was regarded as statistically significant.

## 3. Results

**Figure 1:**
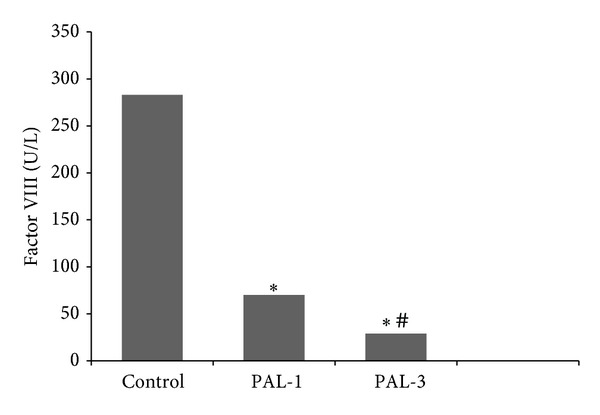
Factor VIII levels in control, PAL-1, and PAL-3 groups. PAL-1 group: receiving paliperidone 1 mg/kg for ten days; PAL-3 group: receiving paliperidone 3 mg/kg for ten days. *P < 0.05 versus control group. ^#^ *P* < 0.05 versus PAL-1 group.

**Figure 2:**
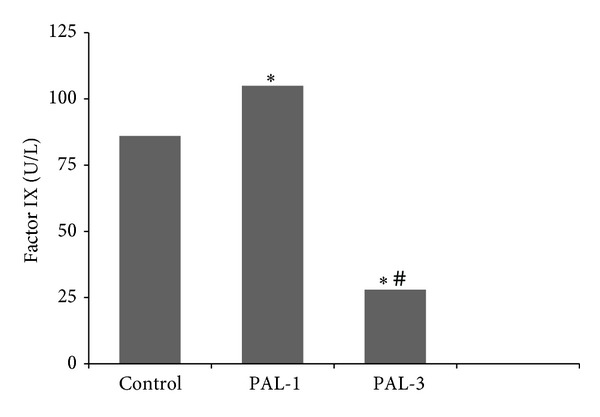
Factor IX levels in control, PAL-1, and PAL-3 groups. PAL-1 group: receiving paliperidone 1 mg/kg for ten days; PAL-3 group: receiving paliperidone 3 mg/kg for ten days. *P < 0.05 versus control group. ^#^ *P* < 0.05 versus PAL-1 group.

Generally there was no statistical difference among groups for hemogram parameters. Hemogram parameters such as white blood cell, red blood cell, and platelet counts did not change in the paliperidone treated groups compared to the controls. Hct values in PAL-1 rats were higher compared to the PAL-3 group only, which is presented in Table 1. There was no significant difference between the groups in terms of hemostatic parameters except factors VIII and IX (Table 2). Factor VIII levels decreased in the PAL-1 and PAL-3 groups, and this decrease was significantly greater in the PAL-3 group (Table 2, Figure 1). The levels of factor IX in PAL-1 and PAL-3 groups were significantly different compared to control and it was higher in PAL-1 than PAL-3. The levels of factor IX decreased in PAL-3 rats, but its levels also increased in PAL-1 rats compared to the control group (Table 2, Figure 2).

## 4. Discussion

In paliperidone palmitate, treated female rats were not seem any abnormal changes in hemogram parameters at the end of ten days. In FDA application files, it has been reported that paliperidone palmitate did not cause any changes in hematology parameters in female rats at 10 mg eq/kg/month during 104 weeks. In contrast, there were statistically significant differences such as decrease in Hct, Hb, and RBCs in female and male Wistar rats and increase in MCH and MCHC in male rats [9]. Most of the parameters of anticoagulation and coagulation except factors VIII and IX were not changed in the present study. There are no studies which investigated the relationship between paliperidone and coagulation in the literature. We reviewed antipsychotic drugs which are in the same group with paliperidone in order to interpret our findings with paliperidone on coagulation.

Numerous studies show a relationship between the use of antipsychotic drugs and venous thromboembolism. The most reported drug for venous thromboembolism during treatment with antipsychotic drug is clozapine [1]. Clozapine and its metabolite N-desmethyl clozapine increased 5-HT-induced platelet aggregation, and N-desmethyl clozapine increased aggregation in lowest doses, but inhibited at the highest doses [1]. Olanzapine has been also related to venous thromboembolism reported in previous case reports [5, 10]. The laboratory abnormalities (thrombocytopenia) in 5% of 26 adult cases of an acute olanzapine poisoning were found [11]. The aPTT was weakly but significantly shortened by clozapine and olanzapine [1]. Axelsson et al. have not found any solid clinical evidence suggesting such platelet effects of olanzapine. In contrast, olanzapine inhibited platelet [1]. Carrizo et al. reported that taking typical antipsychotic group had significantly higher AT-III levels, but fibrinogen levels did not differ between the groups: they suggested that the observed abnormalities were not related to a direct drug effect (olanzapine) [12].

The Medline database reviewed a total of 438 reported for venous thromboembolic events with clozapine, risperidone (283), and olanzapine (241): it has been displayed as an evidence-based that antipsychotic drugs increase the risk of venous thromboembolic events [13]. However, some studies do not support this data. For example, risperidone and 9-OH-risperidone did not activate human platelets, or plasma coagulation, nor did they inhibit whole blood plasma coagulation or fibrinolysis *in vitro* studies [2]. The findings of *in vitro* experiments did not support a thrombogenic effect of risperidone or its metabolite on human platelet function, plasma coagulation, or fibrinolysis. Also, there is no evidence for a relationship between endothelial dysfunction and using an atypical antipsychotic olanzapine, clozapine, risperidone, or paliperidone [14]. Results propose a portion of venous thromboembolic events in patients with acute psychosis may be associated with the pathogenic mechanisms of psychosis instead of antipsychotic drug treatment. For a safe antipsychotic treatment it is necessary to find the exact cause of venous thromboembolism in psychotic patients [15].

Paliperidone disrupted the serum levels of some coagulation factors in the present study: this event suggests that the drug may affect the liver. It is well known that risperidone can lead to drug-induced hepatitis in patients [16]. Indeed, the chronic treatment with risperidone or paliperidone induced liver enzymes such as fatty acid desaturase 2 [17]. On the other hand, paliperidone is not significantly metabolized in the liver according to the results of pharmacokinetic studies [18]. In paliperidone palmitate treated animals, liver and renal function tests were not clinically significant [8]. Paliperidon is excreted by kidney without the need for liver metabolism. Paliperidone shows minimal drug-drug interaction and thus paliperidone may be safer to be used in patients with serious hepatic disease or when taking other medications [19]. Further, risperidone induced hepatic abnormalities completely remitted after switching to paliperidone [20]. Based on these studies, paliperidone may be well tolerated in schizophrenia or schizoaffective disorder patients with liver disease [21].

According to the literature, the antipsychotic drug risperidone is able to lead to both thromboembolism and bleeding. It has been pointed out in some studies bleeding that is associated with risperidone. The use of antipsychotic drugs was associated with an increased risk of gastrointestinal and intracranial bleeding [22]. Researchers reported that risperidone is associated with gastrointestinal bleeding and nose bleeds in patients with different age groups [23, 24]. Indeed, paliperidone dose-dependently decreased the levels of factor VIII in our study and on the other hand, paliperidone increased the levels of factor IX at dose of 1 mg/kg/day and decreased it at dose of 3 mg/kg/day. According to our results, paliperidone has a character of trigger both clotting and bleeding in dose irrelevant. FDA application file also reported that drug related changes in hematology were not dose-dependent [9].

Paliperidone has led to changes in the serum levels of coagulation factors VIII and IX in rats. As a result, paliperidone may be causing thromboembolism or bleeding in a dose-independent manner. We suggest that clinicians should be vigilant against unexpected bleeding or embolism in patients using paliperidone.

## Figures and Tables

**Table 1:** The hemogram parameters in control, PAL-1, and PAL-3 groups.

	Control (*n* = 8)	PAL-1 (*n* = 7)	PAL-3 (*n* = 7)
WBC (×10^3^/L)	7.38 ± 1.49	6.14 ± 1.05	7.72 ± 3.96
RBC (×10^6^/L)	7.31 ± 0.76	7.33 ± 0.47	6.85 ± 0.43
Hgb (g/dL)	13.90 ± 0.50	13.71 ± 0.75	14.05 ± 0.86
Hct (%)	40.56 ± 2.69	43.22 ± 2.24*	40.00 ± 2.69^#^
MCV (fL)	55.80 ± 4.79	58.97 ± 1.94	58.37 ± 1.28
PLT (×10^3^/L)	990 ± 285	810 ± 34	926 ± 175
MPV (fL)	6.65 ± 1.40	6.20 ± 0.32	6.17 ± 0.17

PAL-1 group: receiving paliperidone 1 mg/kg for ten days; PAL-3 group: receiving paliperidone 3 mg/kg for ten days. Values are expressed as means ± SD.

*P < 0.05 versus control group; ^#^ *P* < 0.05 versus PAL-1 group.

**Table 2:** Hemostatic parameters in control, PAL-1, and PAL-3 groups.

	Control (*n* = 8)	PAL-1 (*n* = 7)	PAL-3 (*n* = 7)
PT (sec)	12.30 ± 0.40	12.58 ± 0.43	12.14 ± 0.44
INR (%)	0.98 ± 0.03	1.00 ± 0.03	0.96 ± 0.04
aPTT (sec)	14.22 ± 0.73	14.32 ± 0.74	14.54 ± 0.54
Fibrinogen (mg/dL)	212 ± 115	254 ± 217	178 ± 44
Factor II (U/L)	74 ± 6	74 ± 4	74 ± 6
Factor VIII (U/L)	283 ± 47	70 ± 23*	29 ± 7^∗#^
Factor IX (U/L)	86 ± 8	105 ± 15*	28 ± 5^∗#^
Factor X (U/L)	63 ± 9	56 ± 7	63 ± 5
AT-III (%)	170 ± 36	137 ± 41	160 ± 36
Protein C (*μ*g/mL)	1.26 ± 0.95	1.84 ± 1.19	1.07 ± 0.89
Protein S (*μ*g/mL)	23.25 ± 1.58	22.42 ± 0.53	23.42 ± 0.97

PAL-1 group: receiving paliperidone 1 mg/kg for ten days; PAL-3 group: receiving paliperidone 3 mg/kg for ten days. Values are expressed as means ± SD.

*P < 0.05 versus control group; ^#^ *P* < 0.05 versus PAL-1 group.
